# Cardiac arrest complicating routine throat pack insertion in oral and maxillofacial surgery under general anesthesia: A case report

**DOI:** 10.3892/mi.2026.301

**Published:** 2026-02-11

**Authors:** Kana Yamada, Makoto Adachi

**Affiliations:** 1 Department of Anesthesiology, Nagoya Tokushukai General Hospital, Kasugai, Aichi 487-0016, Japan; 2 Department of Oral and Maxillofacial Surgery, Nagoya Tokushukai General Hospital, Kasugai, Aichi 487-0016, Japan

**Keywords:** throat pack, cardiac arrest, vagal reflex, oral surgery, mandibular third molar

## Abstract

Throat packs are routinely inserted during oral and maxillofacial surgery and are generally considered safe. However, severe vagally mediated cardiovascular reflexes can rarely occur during airway or oropharyngeal manipulation. The present study describes a rare case of cardiac arrest directly attributed to throat pack insertion. A healthy 54-year-old male patient underwent third molar extraction under general anesthesia. Immediately following throat pack placement, he developed sudden cardiac arrest with complete electrical asystole lasting ~20 sec. Spontaneous circulation returned rapidly following the intravenous administration of ephedrine (8 mg) without the need for chest compressions. This event was most consistent with a vagal reflex-mediated response. The present case report indicates that vigilant hemodynamic monitoring, adequate ventilation and appropriate anesthetic depth are essential even during routine adjunctive steps, such as throat pack insertion.

## Introduction

Sudden cardiac arrest during non-cardiac surgery is a rare, yet potentially fatal complication. In head and neck procedures, neurally mediated reflexes including direct vagal stimulation, trigeminocardiac reflex and oculocardiac reflex can trigger severe cardiovascular events (1).

Throat packs are commonly used during oral and maxillofacial surgery to prevent aspiration, although their efficacy remains a matter of debate (2). While generally considered safe, cardiac complications from airway manipulation have been increasingly recognized. Direct laryngoscopy can cause severe bradycardia and asystole through vagal stimulation (3), and even jaw thrust maneuvers, typically used for simple airway maintenance, can trigger profound vagal responses leading to asystole (4). Given this context, throat pack insertion, though considered routine practice, may pose a similar risk of severe vagally mediated cardiac events. The trigeminovagal reflex, with an incidence of ~~1.6% in maxillofacial surgical procedures, has been reported during mouth opening with dental mouth gags, although such cases resulting in asystole are extremely rare (5).

The present study describes a rare case of transient cardiac arrest directly attributed to throat pack insertion during oral surgery. In addition, the present study provides a discussion of the underlying neurophysiological mechanisms and clinical implications for routine maxillofacial procedures.

## Case report

An otherwise healthy 54-year-old male patient (ASA Physical Status 1; height, 173 cm; weight, 78.0 kg) presented to Nagoya Tokushukai General Hospital (Kasugai, Japan) and was scheduled for a mandibular horizontal impacted third molar extraction under general anesthesia.

**Figure 1:**
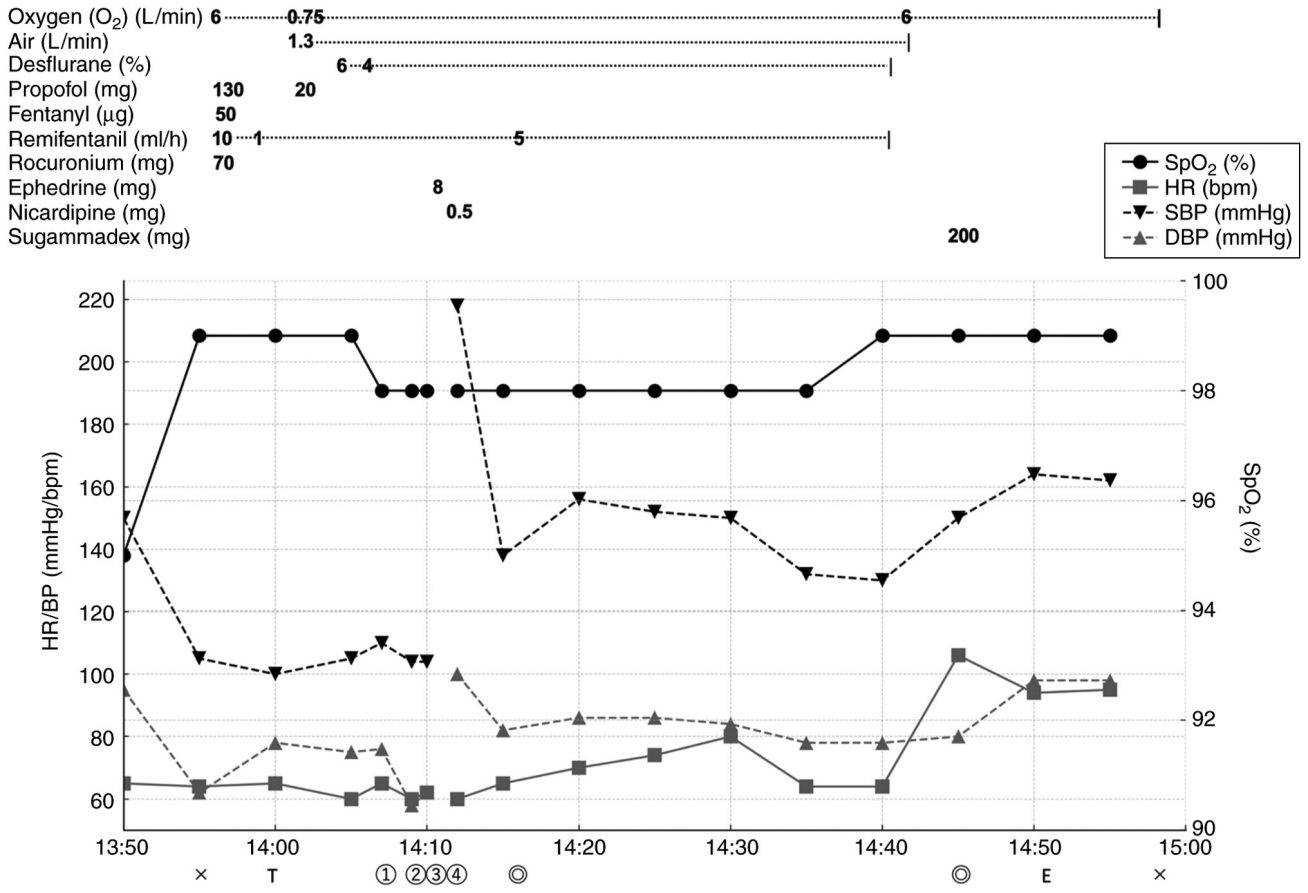
Intraoperative anesthetic chart demonstrating the timeline of anesthetic management, including medication administration and the occurrence of cardiac arrest immediately following throat pack insertion. The chart illustrates the progression from normal vital signs to cardiac arrest and subsequent recovery following ephedrine administration. Crosses mark the start and end of anesthesia; T, intubation; E, extubation; double circles, indicate the start and end of surgery; circled numerals ①-④ indicate: ① throat pack insertion, ② cardiac arrest, ③ ephedrine (8 mg) administration, ④ return of spontaneous circulation. Heart rate values represent trend-averaged monitor data; transient electrical asystole is therefore depicted as an interruption of the HR trace rather than a numerical value of zero. SpO_2_, peripheral oxygen saturation; HR, heart rate; BP, blood pressure; DBP, diastolic blood pressure; SBP, systolic blood pressure.

Anesthesia was induced with propofol 130 mg (1.67 mg/kg), fentanyl 50 µg (0.64 µg/kg), and rocuronium 70 mg (0.90 mg/kg), followed by successful left nasal intubation using a Ring-Adair-Elwyn (RAE) tube (7.0 mm, 27 cm). Anesthesia was maintained with desflurane and remifentanil infusion (Fig. 1).

Following intubation, throat packing was performed according to standard institutional protocol using sterile moistened gauze (30x30 cm). The gauze was gently placed into the oropharynx using long forceps, positioned around the endotracheal tube without excessive pressure.

**Figure 2:**
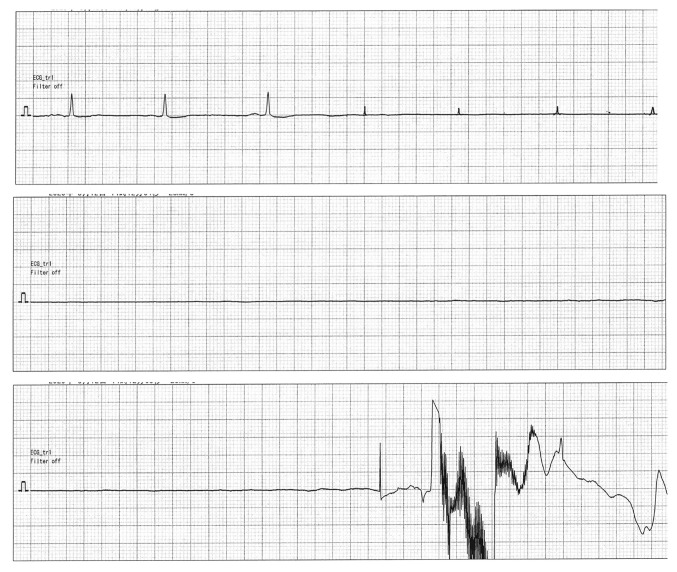
Intraoperative electrocardiographic monitoring findings during throat pack insertion (lead II, paper speed 25 mm/s, gain 10 mm/mV). The three consecutive strips demonstrate: (Upper panel) baseline sinus rhythm before throat pack insertion; (middle panel) complete electrical asystole with absence of all electrical activity; (lower panel) low-amplitude irregular waveform preceding recovery of sinus rhythm after ephedrine administration and stimulus cessation. The asystolic period lasted ~20 sec.

Immediately following throat pack insertion, the patient developed sudden cardiac arrest. Electrocardiographic monitoring revealed complete electrical asystole with absence of all cardiac electrical activity (Fig. 2). The asystolic period lasted ~20 sec, during which the pulse was impalpable.

Upon recognition of profound vagally mediated bradyarrhythmia progressing to electrical asystole, the surgeon immediately ceased all pharyngeal manipulation and removed the throat pack. Ventilation was maintained with 100% oxygen (6 l/min) throughout the episode, and SpO_2_ remained >95%. Given the maintained oxygenation, the absence of pulseless electrical activity confirmed by continuous arterial line monitoring, and the brief duration of the arrhythmia, chest compressions were not initiated. Ephedrine (8 mg) was administered intravenously as the initial vasopressor due to its combined α- and β-adrenergic effects. Atropine was prepared, but was not required, as spontaneous circulation returned ~20 sec following the administration of ephedrine and stimulus cessation. The cardiac rhythm of the patient normalized, and the surgery was completed successfully without further cardiovascular complications. Post-operatively, the patient recovered uneventfully with no evidence of cardiac complications.

On the first post-operative day, the patient was referred to the cardiology department at Nagoya Tokushukai General Hospital for a comprehensive cardiac evaluation. Coronary computed tomography angiography was performed post-operatively (data not shown; images not available) and did not reveal clinically significant coronary stenosis according to the cardiology report. Transthoracic echocardiography demonstrated normal ventricular wall motion and function, supporting the absence of overt structural heart disease. Based on these findings and the clinical course, the event was considered most consistent with a vagally mediated reflex mechanism.

## Discussion

### Reflex arc and anatomical background

Sensory inputs from glossopharyngeal and vagal afferents converge on the nucleus tractus solitarius (NTS) of the medulla, where baroreceptor signals from the carotid sinus, aortic arch, and cardiopulmonary receptors are integrated (6). Parasympathetic efferents arising from the dorsal motor nucleus of the vagus nerve and nucleus ambiguus then project to the sinoatrial and atrioventricular nodes, exerting inhibitory effects on cardiac conduction. Experimental research has further demonstrated that the disruption of NTS neuronal activity directly induces bradycardia and hypotension (7), underscoring the central role of this reflex arc in cardiovascular regulation.

**Figure 3:**
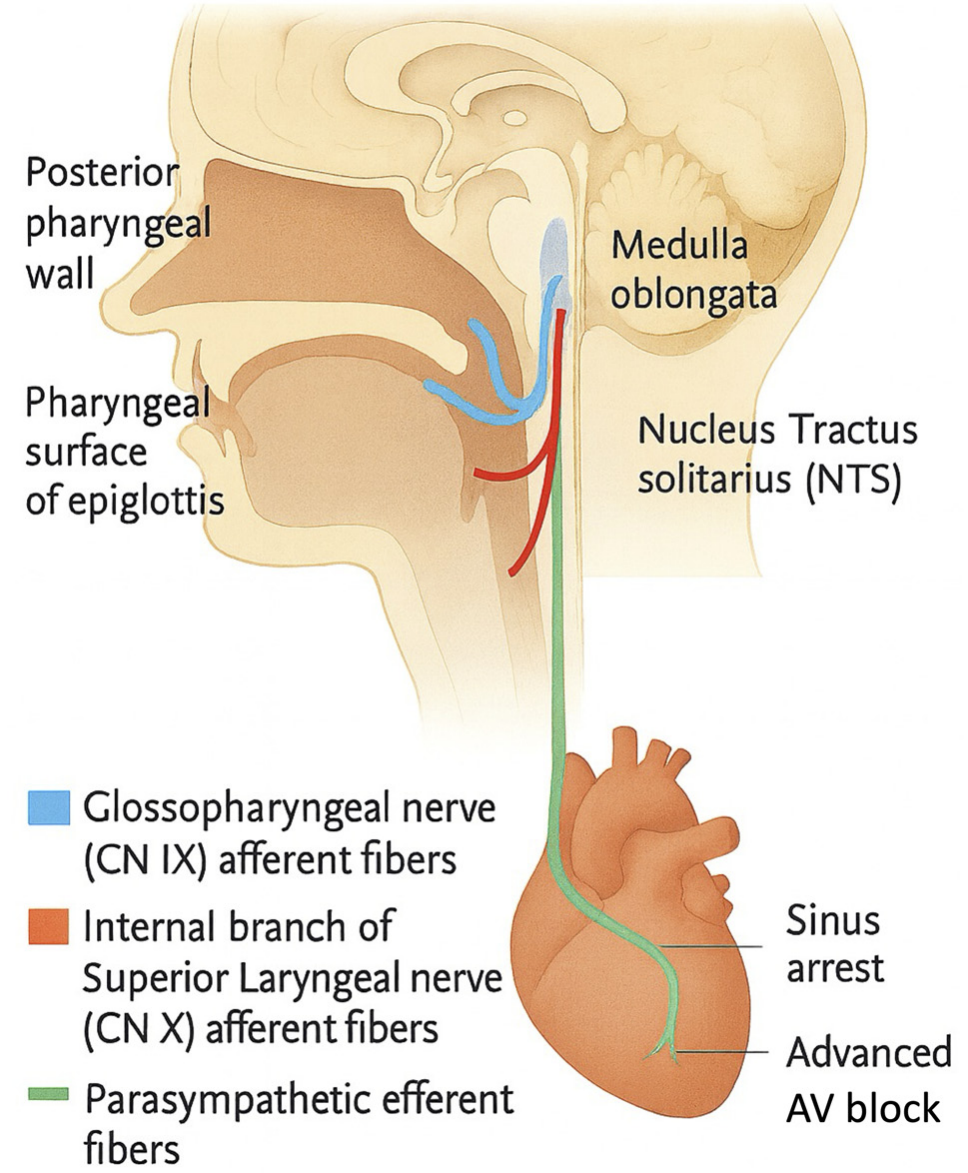
Neural reflex pathway of pharyngeal-stimulation-induced cardiac arrest. Sagittal schematic diagram illustrating sensory afferents from the glossopharyngeal nerve (CN IX, blue; posterior pharyngeal wall) and the internal branch of the superior laryngeal nerve (CN X, red; pharyngeal surface of the epiglottis) entering the nucleus tractus solitarius (NTS, gray) in the medulla separately. Signals from the NTS activate medullary vagal efferent nuclei, the nucleus ambiguus and dorsal motor nucleus, whose parasympathetic fibers (green) project via the vagus to the heart; excessive vagal activation during airway manipulation can precipitate sinus arrest and high-grade atrioventricular block. This illustration was generated using ChatGPT-4 (OpenAI).

During throat pack placement, the stimulation of the laryngopharyngeal mucosa, particularly the epiglottic surface innervated by the internal branch of the superior laryngeal nerve, has been reported to trigger vagal reflexes that may culminate in profound bradyarrhythmia or asystole (3). In addition, the activation of the Bezold-Jarisch reflex, mediated by cardiac receptors with non-myelinated vagal C-fiber afferents, produces a characteristic triad of bradycardia, hypotension and peripheral vasodilation (8). These reflex mechanisms collectively provide a plausible physiologic explanation for the abrupt vagally mediated cardiac arrest observed in the case presented herein (Fig. 3).

### Clinical significance of throat pack-induced cardiac arrest

Reflex-mediated cardiovascular responses may progress stepwise. Campagna and Carter (8) described how vagal reflexes can evolve from initial bradycardia and hypotension to sinus arrest and high-grade atrioventricular block, and ultimately to asystole and pulseless electrical activity. Clinically, such events have been observed during airway manipulation: Redmann *et al* (3) reported asystole during suspension microlaryngoscopy, attributed to direct vagal stimulation, while Chung *et al* (9) documented transient asystole immediately after balloon dilation of the Eustachian tube.

In contrast to these reports of vigorous stimuli, cardiac arrest from throat pack insertion alone remains uncommon. Its occurrence in the case described herein, despite the minimally invasive nature of the intervention, may reflect high baseline vagal tone or a lowered reflex threshold under light anesthesia.

### Risk factors and prevention

Under light anesthesia, the suppression of sympathetic activity predisposes patients to parasympathetic dominance. Propofol and suxamethonium can produce profound bradycardia without anticholinergic premedication (10), while hypercapnia enhances cardiac vagal responsiveness (11). Fluctuations in autonomic balance have also been implicated in vagally mediated bradycardia and circulatory collapse in patients with syncope (12). Adequate anesthetic depth and ventilation before pharyngeal manipulation are therefore essential, and prophylactic atropine may be warranted when vagal predominance is anticipated.

### Clinical management

In the patient described in the present case report, spontaneous cardiac rhythm resumed within ~20 sec without chest compressions after cessation of the stimulus and the administration of 8 mg ephedrine. This rapid recovery is consistent with vasovagal cardiac arrest, in which vagal dominance and sympathetic inhibition predominate (8,12). The prompt return of spontaneous circulation suggests that pharmacologic intervention effectively interrupted the reflex arc. Clinically, the key measures are to discontinue the provoking stimulus immediately and administer anticholinergic or vasopressor agents without delay.

While the temporal association and clinical course strongly suggest a vagally mediated mechanism, definitive confirmation would require comprehensive exclusion of alternative factors including anesthetic depth, ventilation status and baseline autonomic tone, data not completely documented in real-time during this event.

### Limitations

The present case report has several limitations which should be mentioned. First, as a single case report, the findings cannot be generalized to all patients undergoing throat pack insertion. Second, although coronary computed tomography angiography was performed, the original axial and multiplanar reconstructed imaging data were unavailable for publication, which limits independent visual assessment of coronary anatomy. Nevertheless, the cardiology report, normal transthoracic echocardiographic findings and the uneventful postoperative course support the absence of clinically significant structural heart disease. Third, other potential contributing factors that may have predisposed this patient to vagal reflex, such as individual variations in autonomic tone or unrecognized cardiovascular sensitivity, cannot be definitively excluded.

In conclusion, the exact mechanism of reflex activation remains speculative, as direct neurophysiological monitoring was not performed during the procedure, and long-term follow-up data are limited, although no immediate complications were observed.

## Data Availability

The data generated in the present study may be requested from the corresponding author.
